# Synthesis, Structural and Thermal Studies of 3-(1-Benzyl-1,2,3,6-tetrahydropyridin-4-yl)-5-ethoxy-1*H*-indole (D2AAK1_3) as Dopamine D_2_ Receptor Ligand

**DOI:** 10.3390/molecules23092249

**Published:** 2018-09-04

**Authors:** Magda Kondej, Agata Bartyzel, Monika Pitucha, Tomasz M. Wróbel, Andrea G. Silva, Dariusz Matosiuk, Marián Castro, Agnieszka A. Kaczor

**Affiliations:** 1 Department of Synthesis and Chemical Technology of Pharmaceutical Substances with Computer Modeling Laboratory, Faculty of Pharmacy with Division of Medical Analytics, Medical University of Lublin, 4A Chodźki St., PL-20093 Lublin, Poland; magda.kondej@onet.pl (M.K.); tomasz.wrobel@umlub.pl (T.M.W.); dariusz.matosiuk@umlub.pl (D.M.); 2 Department of General and Coordination Chemistry, Maria Curie-Skłodowska University, M. Curie-Skłodowskiej Sq. 2, PL-20031 Lublin, Poland; agata.bartyzel@poczta.umcs.lublin.pl; 3 Independent Radiopharmacy Unit, Department of Organic Chemistry, Faculty of Pharmacy with Division of Medical Analytics, Medical University of Lublin, 4A Chodźki St., PL-20093 Lublin, Poland; monika.pitucha@umlub.pl; 4 Department of Drug Design and Pharmacology, Faculty of Health and Medical Sciences, University of Copenhagen, 2100 Copenhagen Ø, Denmark; 5 Department of Pharmacology, Universidade de Santiago de Compostela, Center for Research in Molecular Medicine and Chronic Diseases (CIMUS), Avda de Barcelona, E-15782 Santiago de Compostela, Spain; andrea.garcia.silva@rai.usc.es (A.G.S.); marian.castro@usc.es (M.C.); 6 School of Pharmacy, University of Eastern Finland, Yliopistonranta 1, P.O. Box 1627, FI-70211 Kuopio, Finland

**Keywords:** dopamine D_2_ receptor, dopamine D_2_ receptor antagonist, molecular modeling, thermal studies, X-ray studies

## Abstract

Compound D2AAK1_3 was designed as a modification of the lead structure D2AAK1 (an in vivo active multi-target compound with nanomolar affinity to a number of aminergic GPCRs) and synthesized in the reaction of 5-ethoxyindole and 1-benzyl-4-piperidone. This compound has an affinity to the human dopamine D_2_ receptor with K_i_ of 151 nM. The aim of these studies was the structural and thermal characterization of the compound D2AAK1_3. In particular; X-ray studies; molecular docking and molecular dynamics as well as thermal analysis were performed. The studied compound crystallizes in orthorhombic system; in chiral space group *P*2_1_2_1_2_1_. The compound has a non-planar conformation. The studied compound was docked to the novel X-ray structure of the human dopamine D_2_ receptor in the inactive state (PDB ID: 6CM4) and established the main contact between its protonatable nitrogen atom and Asp (3.32) of the receptor. The obtained binding pose was stable in molecular dynamics simulations. Thermal stability of the compound was investigated using the TG-DSC technique in the air atmosphere, while TG-FTIR analyses in air and nitrogen atmospheres were also performed. The studied compound is characterized by good thermal stability. The main volatile products of combustion are the following gases: CO_2_; H_2_O toluene and CO while in the case of pyrolysis process in the FTIR spectra; the characteristic bands of NH_3_; piperidine and indole are additionally observed.

## 1. Introduction

Dopamine receptors belong to rhodopsin-like, aminergic G protein-coupled receptors (GPCRs) and play a crucial role in the central nervous system. There are five subtypes of dopamine receptors (D_1_, D_2_, D_3_, D_4_ and D_5_) which can be divided into the two groups D_1_-like (D_1_ and D_5_) and D_2_-like (D_2_, D_3_ and D_4_) depending on activation or inhibition of a secondary messenger, cyclic adenosine monophosphate (cAMP) [1]. Dopamine receptors are important for the pathomechanism of Parkinson’s disease [2], Huntington’s disease, schizophrenia [3], depression, bipolar disorder, sexual disorders, dementia etc. [4]. As a consequence, the dopaminergic system has been useful for over a half of century for the pharmaceutical industry, which resulted in a number of dopaminergic prodrugs, agonists, antagonists and enzyme inhibitors being involved in the synthesis or metabolism of dopamine.

In particular, targeting the dopamine D_2_ receptors is an established approach for the treatment of schizophrenia, which is in accordance with dopaminergic hypothesis of this disease [5]. First, second and third generation antipsychotics are currently available on the market and all of these are either dopamine D_2_ receptor antagonists (with additional affinity to other dopamine receptor subtypes as well as to serotonin receptors, in particular 5-HT_2A_ receptors) or partial agonists/biased ligands of the dopamine D_2_ receptor.

Arylpiperazines [6,7,8] and to the lesser extent arylpiperidines [9,10] and aryltetrahydropyridines [9] have been reported as privileged structures for aminergic GPCRs, in particular serotonin and dopamine receptors. Moreover, aryltetrahydropyridines are known as ligands of other targets, e.g., σ receptor ligands [11] as well as peroxisome proliferators-activated receptors (PPARs, belonging to nuclear receptor family) agonists with antidiabetic activity and monoamine oxidase B (MAO-B) inhibitors [12,13].

**Figure 1:**
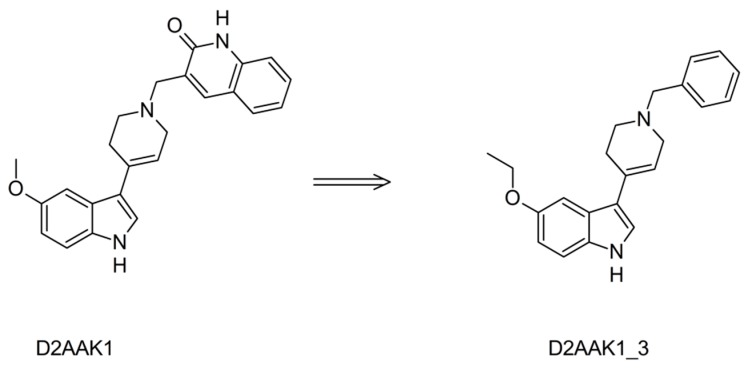
The investigated compound D2AAK1_3 as a modification of the lead structure D2AAK1.

In the search for novel potential antipsychotics, we performed structure-based virtual screening aimed at identifying dopamine D_2_ receptor antagonists [14]. We identified 10 potentially interesting compounds and four of them, i.e., D2AAK1 possessing aryltetrahydropyridine scaffold [15], D2AAK2, D2AAK3 and D2AAK4 were subjected to detailed in vitro, in silico and in vivo investigation. In particular D2AAK1 (Figure 1) is a promising multi-target lead structure with nanomolar affinity to a number of dopamine and serotonin receptors with antipsychotic, anxiolytic and procognitive activity in vivo [15]. We designed a modification of D2AAK1, i.e., D2AAK1_3 (Figure 1) and performed detailed experimental and computational structural as well as thermal studies for this compound. The rationale of the presented study can be summarized as follows: (1) urgent need to design more efficient and safer antipsychotics; (2) promising in vitro and in vivo results for the lead structure D2AAK1; (3) describing ligand-receptor interactions at the molecular level to design more potent compounds; (4) significance of ligand X-ray structure to search for its bioactive conformation and (5) importance of thermal studies for characterization of the compound as a potential drug.

## 2. Results and Discussion

### 2.1. Chemistry

**Scheme 1:**
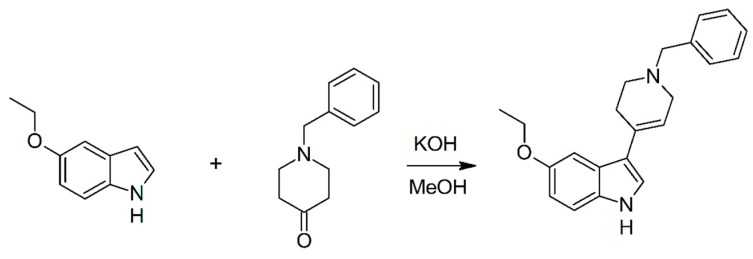
Synthesis of D2AAK1_3.

Compound D2AAK1_3 was synthesized from 5-ethoxyindole and 1-benzyl-4-piperidone in methanol/KOH (see Scheme 1) following previously reported methodology [16].

Compound D2AAK1_3 is a new compound and it is an analogue of known 3-(1-benzyl-1,2,3,6-tetrahydropyridin-4-yl)-1*H*-indole and 3-(1-benzyl-1,2,3,6-tetrahydropyridin-4-yl)-5-methoxy-1*H*-indole. 3-(1-Benzyl-1,2,3,6-tetrahydropyridin-4-yl)-1*H*-indole was reported as 5-HT_1A_ and 5-HT_2A_ receptor ligand (with pKi of 5.00 and 7.81, respectively) [17], intermediate in synthesis of N_1_-arylsulfonyl-3-(1,2,3,6-tetrahydropyridin-4-yl)-1*H*-indole derivatives as 5-HT_6_ receptor antagonists [18], intermediate in synthesis of muscarinic receptor allosteric agents [19] and antimalarial compound [20]. 3-(1-Benzyl-1,2,3,6-tetrahydropyridin-4-yl)-5-methoxy-1*H*-indole was reported as 5-HT_7_ receptor ligand [21] and intermediate in synthesis of muscarinic receptor allosteric agents [19].

In the IR spectra of D2AAK1_3 the medium intense band at 3225 cm^−1^ can be assigned to ν (N-H) vibrations of the pyrrole ring [22]. The band is broad and shifted to lower frequencies which can indicate that those atoms are involved in formation of hydrogen bond. This was confirmed by structural analysis. The bands at 1344 cm^−1^ and 1206 cm^−1^ can be assigned to stretching and deformation vibration of the C-N group in the tetrahydropyridine ring system [23].

### 2.2. Affinity to the Dopamine D_2_ Receptor

**Figure 2:**
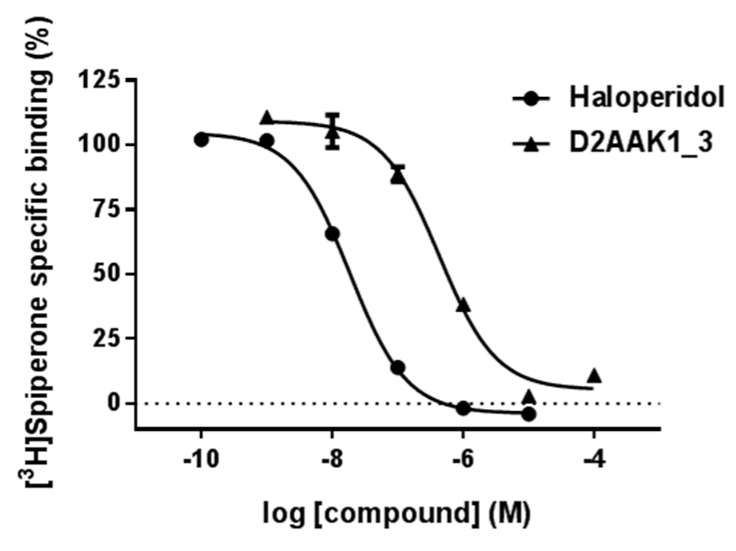
Competition binding curve for D2AAK1_3 at human D_2_ receptors. Displacement of the specific binding of the radioligand [^3^H]Spiperone by D2AAK1_3 in membranes from CHO-K1 cells stably expressing human cloned D_2_ receptors. Haloperidol was used as the reference compound in these assays. The graph shows the results (mean ± SEM) from a single experiment representative of 2 independent experiments performed in duplicate.

D2AAK1_3 fully displaced the specific radioligand binding at D_2_ receptors in a concentration-dependent manner in competition binding experiments (Figure 2). The affinity of D2AAK1_3 to human dopamine D_2_ receptor (*K*_i_) resulting from these experiments was equal to 151 nM (p*K*_i_ = 6.82 ± 0.05).

### 2.3. X-ray Analysis

**Figure 3:**
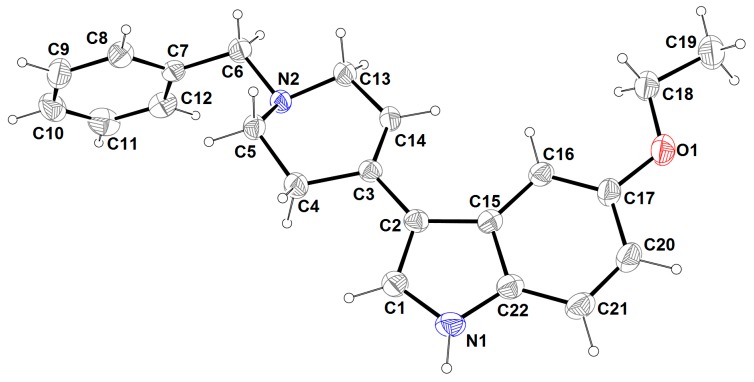
Molecular structures with atom numbering scheme of D2AAK1_3. Displacement ellipsoids are shown at the 30% probability level.

The molecular structure of compound D2AAK1_3 is displayed in Figure 3. The crystallographic and refinement data for the compound are summarized in Table 1, and selected bond distances and angles are listed in Table 2, while hydrogen and π-ring interactions parameters are given in Table 3.

**Figure 4:**
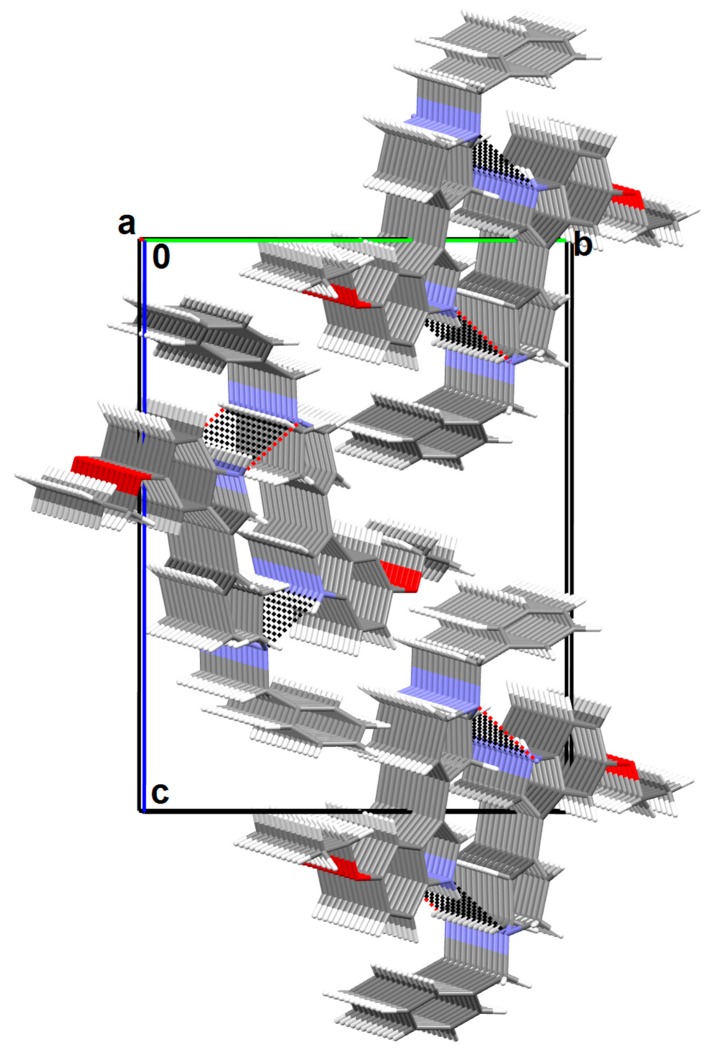
Fragment of the crystal structure of D2AAK1_3 showing formation of one-dimensional columns viewed along the *a*-axis.

The compound crystallizes in noncentrosymmetric space group *P*2_1_2_1_2_1_ with one molecule in an asymmetric unit. The interatomic distances and angles agree with those described in the literature [24] and are comparable with those observed for the other closely related indole derivatives [25,26,27]. The molecule features a disubstituted indole molecule with an ethoxy group connected to a phenyl ring and a 1-benzyl-1,2,3,6-tetrahydropyridin-4yl group linked to a pyrrole ring. The indole ring of the compound is planar with a maximum deviation of 0.019(5) Å of C2 atom from the best plane. The plane formed by O-C-C atoms of ethoxy group is coplanar with the indole moiety (the angle between the two planes is only 0.34(12)°), while the tetrahydropyridine and phenyl rings are significantly rotated (interplanar angles equal 16.37(10)° and 85.57(9)°, respectively). The tetrahydropyridine ring system adopts a half-chair conformation with an N2 atom above (0.326(2) Å) and a C5 atom below (−0.317(3) Å) the mean plane defined by N2/C5/C4/C3/C14/C13 atoms. This is confirmed by the puckering parameters Q = 0.499(2) Å, θ = 49.6(2)° and φ = 27.6(4)° [28,29,30,31]. In the structure, the N1 atom participates in an intramolecular hydrogen bond (N1-H1N···N2^i^, [symmetry code: (i) x − 1/2, −y + 1/2, −z + 1]), as shown in Table 3. As results of these interactions, one-dimensional columns lying parallel to the *a*-axis (Figure 4) are formed. The crystal structure is also stabilized by intermolecular C-H···π interactions (Table 3).

### 2.4. Molecular Modeling

**Figure 5:**
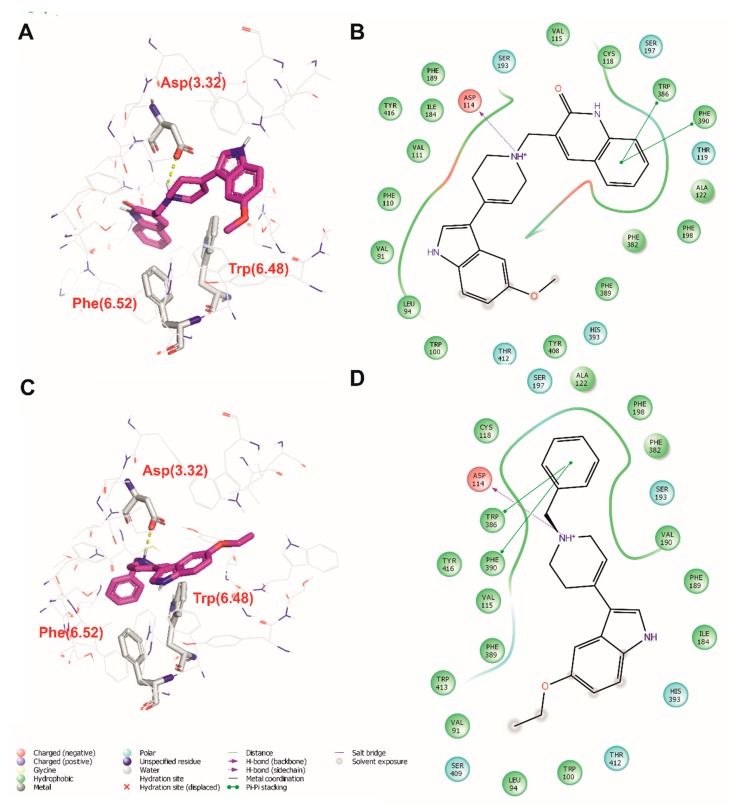
The lead structure D2AAK1 (**A**,**B**) and its derivative D2AAK1_3 (**C**,**D**) in the binding pocket of human dopamine D_2_ receptor. (**A**,**C**) 3D view of the binding site. Ligands represented as sticks with magenta carbon atoms. Protein represented as wire with grey carbon atoms, main interacting residues shown as sticks. Hydrogen bonds shown as yellow dashed lines. Non-polar hydrogen atoms not shown for clarity. (**B**,**D**) 2D view of the binding site.

In order to study ligand-receptor interactions on the molecular level, both the lead structure D2AAK1 and its derivative D2AAK1_3 were docked to the binding pocket of the human dopamine D_2_ receptor (X-ray structure, PDB ID: 6CM4). The docking pose of the lead structure D2AAK1 (Figure 5A,B) is similar to the previously reported docking pose of this compound in the homology model of the D_2_ receptor [14,15]. The main contact of D2AAK1 is the interaction between its protonatable nitrogen atom and Asp114 (3.32) [numbers in brackets are according to Ballesteros-Weinstein nomenclature] [32]. The ligand also forms π-π stacking interactions with Trp386 (6.48) and Phe390 (6.52). The investigated derivative of the lead structure, D2AAK1_3 adopts a similar binding pose to its parent compound, with the benzyl moiety replacing 1,2-dihydroquinolin-2-one scaffold (Figure 5C,D). The positions of alkoxyindole moieties are slightly different for both compounds. The ligand forms the same contacts as the lead structure: Its protonatable nitrogen atom interacts with Asp114 (3.32) and there are π-π stacking interactions with Trp386 (6.48) and Phe390 (6.52). Importantly, the dopamine D_2_ receptor binding site has enough space to accommodate 5-ethoxyindole moiety, which can be important for further optimization of the lead structure.

**Figure 6:**
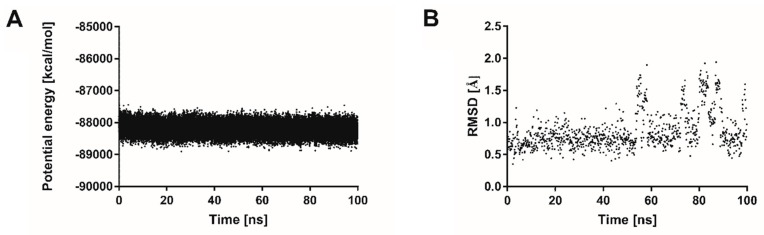
Changes in potential energy (**A**) and ligand RMSD (**B**) during 100 ns molecular dynamics simulations for D2AAK1_3 in complex with human dopamine D_2_ receptor.

100 ns molecular dynamics simulations were performed to investigate the stability of D2AAK1_3 complex with the human dopamine D_2_ receptor. The decreasing value of potential energy (Figure 6A) and ligand RMSD below 2 Å (Figure 6B) indicate that the simulations were performed correctly and the ligand-receptor complex is stable.

**Figure 7:**
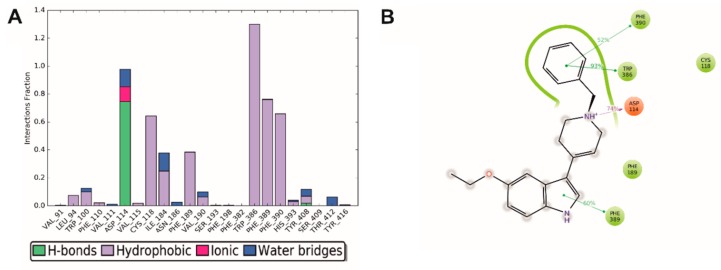
Molecular interactions of D2AAK1_3 with human dopamine D_2_ receptor during 100 ns molecular dynamics simulations. (**A**) histogram of interactions. The stacked bar charts are normalized over the course of the trajectory: For example, a value of 0.7 suggests that 70% of the simulation time the specific interaction is maintained. Values over 1.0 are possible as some protein residue may make multiple contacts of same subtype with the ligand. (**B**) summary of contacts. Interactions that occur more than 30.0% of the simulation time in the selected trajectory (0.00 through 100.00 ns), are shown.

In order to study D2AAK1_3 interactions with the human dopamine D_2_ receptor during 100 ns molecular dynamics simulations, histogram of interactions was generated (Figure 7A). It can be seen that the ligand maintains the interactions with Asp114 (3.32) during 100% of simulations mainly through a hydrogen bond and to the lesser extent, ionic interaction and water-mediated contact. Moreover, hydrophobic contacts are kept between the investigated ligand and the receptor for Trp386 (6.48) during 100% of simulations, Phe389 (6.51) during 70% of simulations, Cys118 (3.36) for 65% of simulations, Phe390 (6.52) during 60% of simulations and Phe189 (5.39) for 40% of simulations. Ile184 from the third extracellular loop is also important for ligand-receptor interactions and maintains hydrophobic contact with the ligand for 30% of simulations and water-mediated interaction for 10% of simulations. Figure 7B summarizes the main ligand-receptor contacts during 100 ns simulations that occur for over 30% of simulations, i.e., Asp114 (3.32), Trp386 (6.48), Phe389 (6.51) and Phe390 (6.52).

### 2.5. Thermal Analysis

**Figure 8:**
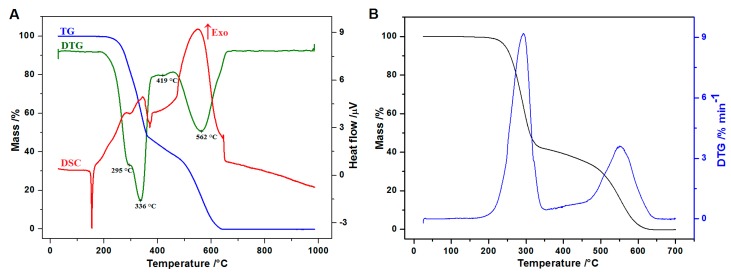
(**A**) TG, DTG and DSC curves of the studied compound recorded in air atmosphere using cylindrical shape crucible; (**B**) TG and DTG curves of the studied compound recorded in air atmosphere using used flat platinum plate (heating rate 10 °C min^−1^).

**Figure 9:**
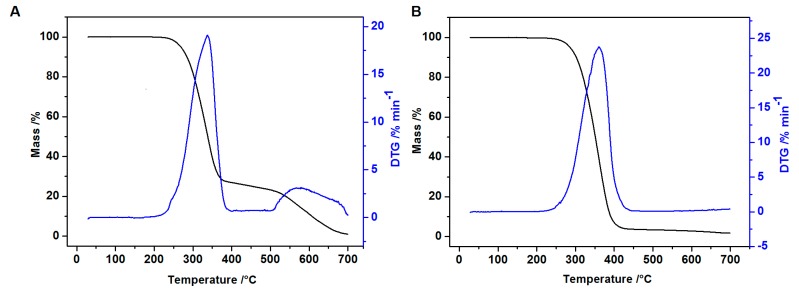
TG and DTG curves of D2AAK1_3 recording during thermal analysis with heating rate 20 °C min^−1^ in air (**A**) and N_2_ (**B**) atmospheres.

Thermal analyses of the investigated compound were performed using the TG-DSC (air) and TG-FTIR (air and nitrogen) techniques (Figure 8 and Figure 9).

**Figure 10:**
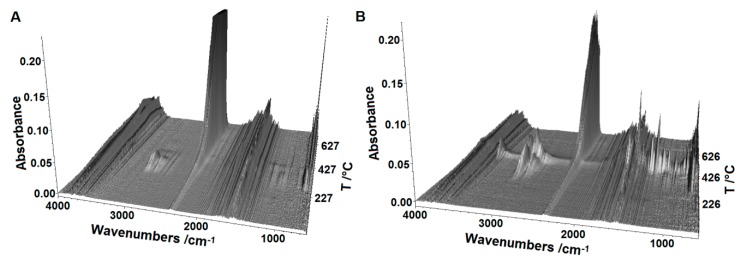
3D diagram of FTIR spectrum of gases evolved during thermal decomposition D2AAK1_3 in air (**A**) and nitrogen (**B**) atmospheres.

The TG-DSC curves providing information about the thermal properties of D2AAK1_3 are shown in Figure 8A. As it can be seen in the TG curves, the compound is stable at room temperature, which is a very important parameter for its potential application as a drug. During the heating, a first change is recorded on the DSC curve as an endothermic peak (*T*_onset_ = 151 °C, T_peak_ = 154 °C). This peak is not accompanied by a mass loss and can be attributed to the melting process. It is sharp, indicating that the compound was synthesized as pure, crystalline substance [22,33]. The enthalpy of fusion calculated from the DSC peak is 25.96 ± 0.93 kJ mol^−1^. Further heating leads to thermal decomposition of D2AAK1_3, which roughly occurs in two stages recorded on a TG curve. The first step of compound thermal degradation is observed at 212–390 °C and 54.43% of the sample is combusted. It is worth mentioning that on the DTG curve, two maxima (at 295 °C and 336 °C) are visible during this stage, but this is not clearly indicated on the TG curve. The formed products are unstable and immediately undergo further decomposition and combustion. The sample fully decomposes at a temperature of approximately 650 °C. In order to better understand the mechanism of the thermal decomposition process, the TG-FTIR analysis of D2AAK1_3 in oxidizing as well as inert atmosphere was performed (see Figure 9). As it can be seen from Figure 9A, the process performed at air atmosphere also occurs in two stages, but the different heating rate (20 °C min^−1^), a larger sample mass and a shape of the crucibles affected the temperature of the decomposition process as well as the mass loss. The first stage starts at 221 °C and as much as 73.30% of sample is combusted. Moreover, in contrast to the thermal decomposition in air atmosphere conducted with heating rate 10 °C min^−1^, here the degradation process is not finished; the residue remaining after the analysis at 700 °C is 2.15%. To confirm this, we conducted a comparative analysis where the same heating rate and similar mass of the sample as in TG-DSC analysis were used (Figure 8B). The course of TG curve was more closely related and all differences in combustion process are probably connected only with the shape of the crucibles. In the case of used flat platinum plate, the decomposition starts earlier (200 °C) and a greater part of the compound (58.41%) decomposed in the first stage compared to the sample heated in a cylindrical shape crucible. The sample is also completely combusted at a temperature of 641 °C. As mentioned earlier together with the thermal analysis of the larger sample mass at air atmosphere the infrared spectroscopy analysis of gaseous products was also performed (Figure 10A). At the beginning of D2AAK1_3 combustion the bands characteristic of water and CO_2_ were recorded. The peaks corresponding to CO_2_ are recorded in the range at 2275–2050 cm^−1^ while the characteristic bands of water molecules, stretching and deformation vibrations, are observed in the range 4000–3450 cm^−1^ and 1950–1300 cm^−1^, respectively [33,34]. Further heating breaks a bond between the benzyl group and the tetrahydropyridine unit. This is confirmed by several bands appearing in the 3200–2800 cm^−1^ range (with four maxima at 3072, 3032, 2933 and 2885 cm^−1^) and 800–600 cm^−1^ (with the maxima at 728 and 692 cm^−1^), which can be assigned to the toluene molecule [35,36].

**Figure 11:**
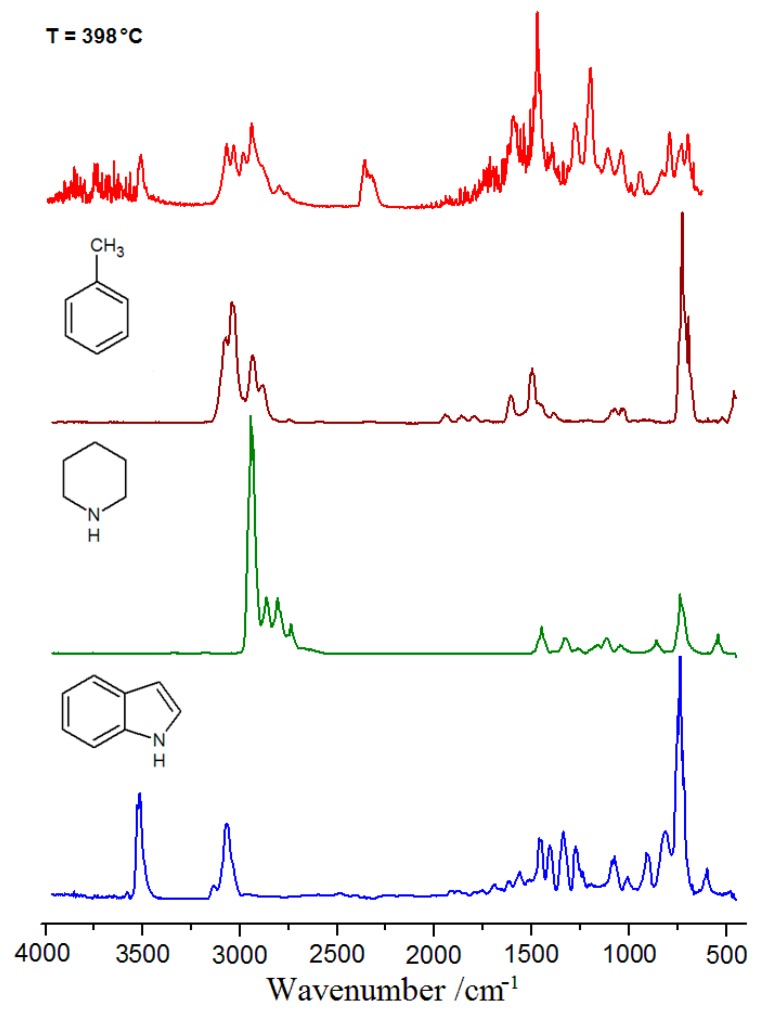
FTIR spectrum of volatile products of thermal decomposition of D2AAK1_3 recorded at 398 °C in inert atmosphere and the spectra of toluene, piperidine and indole.

The bands assigned to C_6_H_5_CH_3_ disappeared at 370 °C. Above this temperature only vibration originated from water and carbon dioxide are observed, indicating that the additional process is mainly related to the combustion of the organic matrix which confirms the presence of an intense exothermic peak on the DSC curve. In contrast to the combustion process, the pyrolysis of D2AAK1_3 undergoes in one stage (Figure 9B) and starts at 230 °C. Similar to analysis conducted in oxidizing atmosphere, at the beginning of the thermal decomposition (about 240 °C) under nitrogen stream in the FTIR spectra of evolved gaseous products, the bands characteristic of H_2_O, CO_2_ and toluene molecules are observed. With the increasing temperature, the intensity of the bands associated with the vibration of C_6_H_5_CH_3_ increases and also appears characteristic of the NH_3_ double peak (965 and 930 cm^−1^) [35,36,37] further indicating a partial degradation of substituent on the pyrrole ring. Around 352 °C in the FTIR spectra of volatile pyrolysis products other fragments of the compound appear, probably in the form of indole and piperidine molecules (see Figure 11). At the end of the pyrolysis process (ca. 430 °C) the peaks characteristic of the CO molecule are observed. This stage is finished at 445 °C and remaining amount of organic matrix equals 3.73%. Further heating above this temperature causes slow pyrolysis of residue and the formation of low mass molecules i.e., CO_2_ H_2_O and CO. The mass loss in the range of 445–470 °C is 2%.

## 3. Materials and Methods

### 3.1. Chemistry

Reactions were routinely monitored by thin-layer chromatography (TLC) on silica gel (60 F_254_ Merck plates) and the products were visualized with ultraviolet light at 254 nm. All NMR spectra were acquired on a Bruker AVANCE III 600 MHz spectrometer equipped with a BBO Z-gradient probe. Spectra were recorded at 25 °C using DMSO as a solvent with a non-spinning sample in 5 mm NMR-tubes. High resolution mass spectra (HRMS) were recorded on a Bruker microTOF-Q II and processed using Compass Data Analysis software. The infrared spectra were recorded on a Thermo Scientific Nicolet 6700 FTIR with a Smart iTR diamond ATR accessory. Data was collected in the range 4000–550 cm^−1^, with a resolution of 4 cm^−1^ for 16 scans.

3-(1-Benzyl-1,2,3,6-tetrahydropyridin-4-yl)-5-ethoxy-1H-indole, D2AAK1_3 5-Ethoxyindole (0.167 g, 1.04 mmol) was dissolved in methanol (10 mL) containing KOH (0.148 g, 2.64 mmol). 0.5 g (2.64 mmol) of 1-benzyl-4-piperidone was added and the reaction mixture was refluxed for 18 h. The mixture was cooled down, poured into water and extracted three times with ethyl acetate. The organic phase was washed with water and dried with MgSO_4_. The drying agent was filtered and the solvent was removed on a rotavap. The remaining solid was crystallized from the mixture of ethanol and diethyl ether (1:1) and washed with diethyl ether. Yield: 46%. ^1^H NMR (600 MHz, DMSO-*d*_6_) δ = 10.96 (d, *J* = 2.1 Hz, 1H, N-H), 7.38–7.31 (m, 5H, Ar-H), 7.29–7.25 (m, 2H, Ar-H), 7.23 (d, *J* = 2.3 Hz, 1H, IndC4-H), 6.75 (dd, *J* = 2.3, 8.8 Hz, 1H, IndC6-H), 6.09–6.02 (m, 1H, C=C-H), 4.01 (q, *J* = 6.9 Hz, 2H, O-CH_2_), 3.58 (s, 2H, Ph-CH_2_), 3.10 (br d, *J* = 3.1 Hz, 2H, N-CH_2_), 2.51 (m, 2H, Pip-CH_2_), 2.65 (t, *J* = 5.7 Hz, 2H, N-CH_2_), 1.32 (t, *J* = 6.9 Hz, 3H, CH_3_); ^13^C NMR (151 MHz, DMSO-*d*_6_) δ = 153.3 (C-O), 139.1 (ArC), 132.5 (IndC), 130.1 (C=C), 129.3 (ArC), 128.7 (ArC), 127.4 (ArC), 125.4 (IndC), 123.8 (IndC), 117.6 (C=C), 116.1 (IndC), 112.7 (IndC7), 112.1 (IndC6), 103.7 (IndC4), 63.9 (O-CH_2_), 62.6 (ArCH_2_), 53.3 (N-CH_2_), 50.4 (N-CH_2_), 29.1 (PipCH_2_), 15.4 (CH_3_). FTIR bands (ν/cm^−1^): 3225(m), 3132(m), 3089(w), 3065(w), 3045(w), 3029(w), 3007(w), 2989(w), 2979(w), 2958(w), 2930(w), 2916(w), 2899(w), 2870(m), 2830(w), 2799(m), 2751(m), 2721(m), 1652(m), 1621(m), 1598(vw), 1578(s), 1528(m), 1496(m), 1483(s), 1464(vs), 1437(m), 1398(m), 1378(w), 1367(m), 1344(m), 1328(m), 1311(m), 1275(vs), 1264(vs), 1250(w), 1206(vs), 1155(w), 1142(m), 1123(s), 1107(vs), 1098(w), 1083(vw), 1062(m), 1046(vs), 1028(s), 1006(w), 991(m), 974(m), 968(m), 951(vs), 940(w), 921(m), 905(m), 896(m), 851(vw), 835(vw), 812(vs), 798(vs), 784(m), 772(s), 760(m), 737(vs), 699(vs), 642(m), 634(m), 610(m), 577(w), 564(w), 560(w). HRMS (ESI): [M + H]^+^ calc. 333.1961; exp. 333.1961.

### 3.2. In Vitro D_2_ Receptor Binding Assay

D_2_ receptor binding assay was performed in membranes from CHO-K1 cells stably expressing the cloned human D_2S_ receptor as previously described [14,15,38]. Competition binding experiments were performed using [^3^H]Spiperone (0.2 nM) as radioligand. Non-specific binding of the radioligand was determined in assay wells containing radioligand and 10 µM sulpiride, and non-specific binding was subtracted from total binding in each assay well to determine radioligand specific binding. D2AAK1_3 was evaluated at increasing concentrations from 10^−9^ to 10^−4^ M. Data analysis was carried out as previously described [38,39].

### 3.3. X-ray Structure Analysis

The single-crystal diffraction measurement was made on an Oxford Diffraction Xcalibur CCD diffractometer with the graphite-monochromated MoK*_α_* radiation. Data sets were collected using the ω scan mode. The program CRYSALIS PRO [40] was used for data collection, cell refinement and data reduction. The data were corrected for Lorentz and polarization effects. A multi-scan absorption correction was applied. The structures were solved by direct methods using SHELXS-2013 and refined by the full-matrix least-squares on F^2^ using SHELXL-2013 [41] (both operating under the WinGX software package) [42]. All non-hydrogen atoms were refined with the anisotropic displacement parameters. The hydrogen atoms residing on carbon atoms were positioned geometrically (C-H = 0.93–0.97 Å) and refined using a riding model with U_iso_(H) = 1.2 or 1.5 U_eq_(C). The N–H hydrogen atom was located in a difference Fourier map and freely refined. The geometrical calculations were performed using the PLATON program (see Supplementary Materials) [43]. In the least-squares refinements, the Flack parameter defined as |F| = (1 − x)|F(+)| + x|F(−)| was refined [44]. The absolute structure parameter is meaningless (0.1(10)) with a rather poor accuracy because the compound is a weak anomalous scatterer (i.e., light atom structure containing only atoms of types O, N, C and H measured with MoKα radiation). For this reason the chemical absolute configuration could not be determined unambiguously. The molecular structures were drawn with ORTEP3 for Windows [45] and Mercury [46]. Crystallographic data were deposited with the CCDC and are available on request, quoting the deposition number CCDC 1855483.

### 3.4. Molecular Modeling

#### 3.4.1. Compound Preparation

X-ray structure of the compound D2AAK1_3 was used as a starting conformation for molecular modeling. The compound was modelled using LigPrep [47] from Schrödinger suite of software (Schrödinger, New York, NY, USA). In order to sample protonation states at the physiological pH, Epik [48] module of Schrödinger suite of software was used.

#### 3.4.2. Molecular Docking

Compounds D2AAK1 and D2AAK1_3 were docked to the novel X-ray structure of the human dopamine D_2_ receptor in the inactive state (PDB ID: 6CM4) [49]. The grid was generated based on co-crystallized ligand, risperidone. Standard precision (SP) method of Glide molecular docking was applied. 20 poses were generated for each compound.

#### 3.4.3. Molecular Dynamics

Molecular dynamics studies of the ligand-receptor complex were performed using Desmond [50] incorporated in Schrödinger suite of software [51] as described previously [52,53,54]. The complex was inserted into POPC membrane, hydrated and ions were added to neutralize protein charges and then to the concentration of 0.15 M NaCl [55]. The complex was minimized and subjected to MD first in the NVT ensemble for 1 ns and then in NPT ensemble for 20 ns with the restrictions on the protein backbone in each case. The production run was performed in NPT ensemble with no restrictions for 100 ns. Analysis of molecular dynamics simulations was performed using the Schrödinger suite of software tools.

### 3.5. Thermal Analysis

Thermal behavior of the investigated compound was determined using Setaram Setsys (Setaram Instrumentation, Ratingen, Germany)16/18 derivatograph, registering TG, DTG and DSC curves. The sample (3.96 mg) was heated in a Al_2_O_3_ crucible between 30 and 1000 °C in flowing air atmosphere (v = 0.75 dm^3^ h^−1^) with a heating rate of 10 °C min^−1^. The temperature and heat flow of the instrument were calibrated by the melting point and enthalpy of indium standard. TG-infrared spectrometry (TG-FTIR) of the title compound was performed using the TGA Q5000 analyzer (TA Instruments, New Castle, DE, USA) interfaced to the Nicolet 6700 FTIR spectrophotometer (Thermo Scientific, Waltham, MA, USA) in oxidizing and inert atmospheres. The samples of 10.65 mg (air atm.) and 19.09 mg (N_2_ atm.) were put in an open platinum crucible and heated from an ambient temperature (~25 °C) to 700 °C with a heating rate of 20 °C min^−1^ (at a flow rate of 25 mL min^−1^). In order to reduce the possibility of gases condensing along the transfer line, the temperature in the gas cell and transfer line were set to 250 and 240 °C, respectively. The FTIR spectra were recorded in the spectral range of 600–4000 cm^−1^ with a resolution of 4 cm^−1^ and 6 scans per spectrum. Thermal decomposition of compound in air atmosphere using TGA Q5000 analyzer was also performed with a heating rate of 10 °C min^−1^ in the range 25–700 °C.

## 4. Conclusions

In summary, we report a novel dopamine D_2_ receptor ligand and characterize it thoroughly using in vitro assay, molecular modeling, X-ray studies and thermal analysis. The investigated compound might be an interesting starting point to design more efficient and safer antipsychotics. Although the studied compound exhibits dopamine D_2_ receptor affinity in the range of 100 nM, some atypical antipsychotics, such as quetiapine, show D_2_ receptor affinity in this range. Moreover, quetiapine is frequently used in spatial conditions such as pregnancy due to its safety profile [56]. Quetiapine appears to produce fewer parkinsonian movement disorders than other atypical antipsychotics (paliperidone, aripiprazole, ziprasidone, risperidone and olanzapine) in schizophrenia [57]. Even clozapine, the gold-standard among atypical antipsychotics, has a D_2_ receptor affinity around this range.

**Scheme 2:**
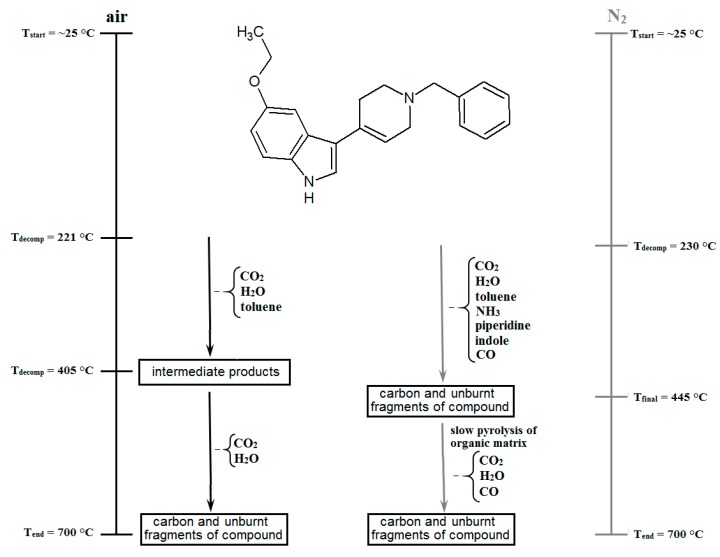
The thermal behavior of compounds in air and N_2_ atmospheres (heating rate 20 °C min^−1^).

The studied compound exhibits high thermal stability which is an important factor for the compound with prospective medical use. The compound melts above 150 °C and decomposes at a temperature higher than 200 °C. In air atmosphere decomposition process of D2AAK1_3 occurs in two stages while in nitrogen the pyrolysis proceeds in one step. The TG-FTIR indicated that the compound has been synthesized as pure and not hygroscopic, as well as that there are no residual solvents in its structure. The analysis also confirmed formation of various compounds during thermal decomposition of D2AAK1_3. The main volatile decomposition products of combustion are the following gases: CO_2_, H_2_O toluene and CO while in the case of pyrolysis process in the FTIR spectra, the characteristic bands of NH_3_, piperidine and indole are additionally observed. The thermal decompositions of D2AAK1_3 in air and nitrogen atmospheres are presented in Scheme 2.

## Figures and Tables

**Table 1:** Crystal data and structure refinement of D2AAK1_3.

Formula	C_22_H_24_N_2_O
Formula weight	332.43
Temperature K	293(2)
Crystal system	orthorhombic
Space group	*P*2_1_2_1_2_1_
a (Å)	5.9600(4)
b (Å)	15.0986(9)
c (Å)	16.1571(9)
Volume (Å^3^)	1814.5(2)
Z	4
Calculated density (g cm^−3^)	1.217
μ (mm^−1^)	0.075
Absorption correction	multi-scan
F(000)	712
Crystal size (mm)	0.50 × 0.20 × 0.20
θ range (°)	2.698 to 27.103
Index ranges	−7 ≤ h ≤ 7 −19 ≤ k ≤ 19 −25 ≤ l ≤ 18
Reflections collected/unique	14,278/3993
R_int_	0.0442
Data/restraints/parameters	3993/0/231
GooF on F^2^	1.000
Final R indices[I > 2σ(I)]	R_1_ = 0.0437, wR_2_ = 0.0887
R indices(all data)	R_1_ = 0.0745, wR_2_ = 0.1019
Largest diff. peak/hole, e Å^−3^	0.156/−0.111

**Table 2:** Interatomic distances and selected bond angles.

***Bond Lengths (Å)***			
C1-N1	1.359(3)	C10-C11	1.364(5)
C1-C2	1.369(3)	C11-C12	1.376(4)
C2-C15	1.441(3)	C13-N2	1.462(3)
C2-C3	1.465(3)	C13-C14	1.497(3)
C3-C14	1.325(3)	C15-C22	1.406(3)
C3-C4	1.506(3)	C15-C16	1.409(3)
C4-C5	1.512(3)	C16-C17	1.373(4)
C5-N2	1.459(3)	C17-O1	1.380(3)
C6-N2	1.472(3)	C17-C20	1.392(4)
C6-C7	1.503(3)	C18-O1	1.416(3)
C7-C8	1.376(3)	C18-C19	1.493(4)
C7-C12	1.383(3)	C20-C21	1.366(4)
C8-C9	1.379(4)	C21-C22	1.386(4)
C9-C10	1.360(4)	C22-N1	1.376(3)
***Bond angles (°)***			
C14-C3-C2	124.6(2)	C3-C14-C13	124.1(2)
C14-C3-C4	118.6(2)	C16-C17-O1	124.3(2)
C2-C3-C4	116.7(2)	O1-C17-C20	114.6(2)
C3-C4-C5	113.0 (2)	C5-N2-C13	108.4(2)
N2-C5-C4	111.0(2)	C5-N2-C6	111.2(2)
N2-C6-C7	114.1(2)	C13-N2-C6	107.7(2)
N2-C13-C14	113.1(2)	C17-O1-C18	118.0(2)
***Torsion angles (°)***			
C4-C5-N2-C13	−65.0(3)	C4-C3-C14-C13	0.6(4)
C3-C4-C5-N2	47.6(3)	N2-C13-C14-C3	−18.2(4)
C14-C3-C4-C5	−15.0(3)	C14-C13-N2-C5	49.2(3)

**Table 3:** Hydrogen bonding and C-H···*Cg* interactions geometry.

***Hydrogen Bond [Å, °]***				
**D-H···A**	**d(D-H)**	**d(H···A)**	**d(D···A)**	∠ **DHA**
N1-H1N···N2 ^i^	0.88(3)	2.41(3)	3.217(3)	154(2)
***C-H···Cg interactions [Å, °]***				
**C-H···*Cg***	**d(D-H)**	**d(H···Cg)**	**d(C···Cg)**	∠ **CH*Cg***
C4-H4B···*Cg*1 ^ii^	0.97	2.94	3.567(3)	124
C19-H19C···*Cg*4 ^iii^	0.96	3.00	3.799(3)	142

Symmetry codes: (^i^) x − 1/2, −y + 1/2, −z + 1; (^ii^) x − 1/2, −y + 1/2, −z + 1; (^iii^) x + 1, y, z; *Cg*1 and *Cg*4 are the centroids of the N1/C1/C2/C15/C22 and C15/C16/C17/C20/C21/C22 rings, respectively.

## Supplementary Materials

The following are available online: cif and checkcif files for the reported compound.
